# Two-Phase Evaluation of the Validity of a Measure for Self-Regulated Learning in Sport Practice

**DOI:** 10.3389/fpsyg.2018.02641

**Published:** 2018-12-21

**Authors:** Lindsay McCardle, Bradley W. Young, Joseph Baker

**Affiliations:** ^1^School of Human Kinetics, University of Ottawa, Ottawa, ON, Canada; ^2^School of Kinesiology and Health Science, York University, Toronto, ON, Canada

**Keywords:** self-regulation, deliberate practice, expert development, metacognition, motivation

## Abstract

Given the potential role of self-regulated learning (SRL) for enhancing practice and expertise development, we aimed to advance a valid and reliable athlete self-report measure of SRL for sport practice. We built on Toering et al. (2012a) initial SRL instrument along with Bartulovic et al. (2017) sport-specific modifications, and created new items to extend the conceptual breadth of the subscales. With a multi-sport sample of 482 athletes (*M*age = 26.45, *SD* = 12.66; 55% female), two analytic phases tested (1) the factorial validity of the initial and the extended inventories, and (2) criterion validity, by examining how SRL scores distinguished skill groups ranging from local to international competitive levels. In Phase 1, the initial measurement model demonstrated psychometric concerns and we opted to pursue a refined model. The extended model demonstrated acceptable factorial validity but resulted in the fewest subscales. In Phase 2, subscales scores from all three models generally distinguished international-level senior (18 + years) athletes from lesser-skilled groups. Integrating the psychometric evidence and between-group effects across the initial, refined, and extended models, we conclude that the refined inventory, the Self-Regulated Learning for Sport Practice (SRL-SP) survey, is the preferred instrument.

## Introduction

Self-regulated learning (SRL), how athletes manage themselves and efforts/activities in learning contexts, refers to a set of psychological processes that are expected to contribute to optimal conditions for sport practice (Tedesqui and Young, 2015). The enactment of SRL self-processes is considered a contributing factor to the development of sport expertise (McCardle et al., 2017). To better understand the interplay between quality sport practice and skill acquisition trajectories (Young and Baker, 2017), research requires valid and reliable measures of SRL. Much of the SRL research in the sport expertise field has relied on athlete self-report surveys. We aimed to advance this research by testing the reliability, factorial, and criterion validity of a scale that assesses facets of SRL that may pertain to expert development.

Self-regulated learners are intentional, strategic, and persistent. SRL focuses on *how* learners actively manage their own learning via planning, monitoring, and adapting sub-processes (Winne and Hadwin, 1998; Zimmerman, 1998, 2000). Varied perspectives on SRL share at least four important assumptions (Pintrich, 2000): (a) learners are active agents in their learning processes; (b) learners can control their cognition, motivation, behavior, and some aspects of the environment; (c) learners hold goals or criteria that direct action and form the basis of metacognitive judgments; (d) SRL processes mediate the relation between achievement outcomes and personal and contextual characteristics. Conceptual models of SRL draw heavily on (a) metacognition, as learners are able to regulate when they are aware of and control their cognitions; and (b) motivation, as engagement in metacognitive and behavioral control is effortful (Zimmerman, 2011).

Conceptually, SRL is a collection of metacognitive and motivational sub-processes that interact dynamically and recursively. Theorists often model SRL using a temporal and/or cyclical framework to provide structure to these sub-processes; for example, Zimmerman (1998), Zimmerman (2000) socio-cognitive model delineates sub-processes occurring before (forethought phase), during (performance phase), and after (reflection phase) a learning task. Evaluation and reflection sub-processes occurring after the task feed forward into planning and goal setting sub-processes for subsequent task efforts. Although many sub-processes have been identified, six have been the particular focus in sport research involving athlete self-report (Toering et al., 2012a). They include *planning* (when learners decide on an approach for strategically accomplishing a task and pursuing goals) *self-monitoring* (when learners track their progress during task engagement), *evaluation* (when learners compare their progress in a session to their standards), *reflection* (when learners look back on progress over multiple sessions to gain insight for future learning), *effort* (learners' proclivity to give mental/ physical exertion), and *self-efficacy* (learners' beliefs that they are able to successfully complete a task).

The earliest survey to gain traction among expertise researchers was the Self-Regulated Learning—Self-Report Scale (SRL-SRS; Toering et al., 2012a). The researchers adopted several scales from education and non-sport domains, compiled them within the same inventory, and submitted them to preliminary validation studies. Toering et al. (2012a) tested 50 items on two samples of 600 adolescents; 46 items were retained on six subscales (planning, self-monitoring, evaluation, reflection, effort, self-efficacy), showing acceptable structural fit, test-retest and internal reliability. Toering et al. (2011) also demonstrated significant correlations between SRL subscales and observed practice behaviors, providing evidence for criterion validity. Researchers (Toering et al., 2009, 2012b; Jonker et al., 2010) also demonstrated differences in subscale scores between more-expert and less-expert athletes; however, differences were most consistently evidenced for reflection, but inconsistently for other subscales. Although this work founded a self-report SRL measure (Toering et al., 2012a), the SRL-SRS (a) is too domain-general (Toering et al., 2013; Bartulovic et al., 2017) and (b) leaves several SRL sub-processes underrepresented. Moreover, it remains to be seen whether it can reliably and validly distinguish more-expert from less-expert groups on key criteria for expertise development.

First, SRL assessment needs to be domain-specific for sport practice. Toering et al. (2012a) conceptualized SRL “as a relatively stable attribute in multiple learning domains” (p. 25). Thus, their SRL-SRS included items addressing regulation of academic (i.e., classroom based) problem solving and mathematics task solutions in addition to more general items. This may be problematic for several reasons. First, conceptual models and evidence suggest learners adjust their approach depending on the context and the specific task (e.g., Cleary and Zimmerman, 2001). Second, principles of concordant measurement suggest that situational behavior (e.g., practice tasks) should be more closely associated with the report of domain-specific and particularly task-specific self-processes. Third, psychological mechanisms underpinning expert sport skills are highly domain-specific (Loffing et al., 2012; Baker and Young, 2014). This literature suggests SRL measurement should be assessed using items that are more specific to the sport practice domain.

In line with this reasoning, Bartulovic et al. (2017) used a vetting process with nine SRL researchers and modified 48 items from Toering et al.'s (2012a) initial pool to be specific to sport practice tasks. They failed to show acceptable structural fit for their modified measurement model; exploratory factor analyses resulted in the removal of multiple poor-fitting items, with a final 31-item model with six subscales matching Toering et al.'s subscales. These new subscales demonstrated impressive criterion validity in relation to skill level (Bartulovic et al., 2017) in that enactment of planning, self-monitoring, effort, and self-efficacy sub-processes explained significantly greater odds of being in an elite skill group than in a less-elite and competitive recreational group. Results also demonstrated that overall SRL reported by athletes (i.e., a score representing the average of all subscales) also distinguished international-level athletes from less-skilled cohorts. The adaptation of the SRL-SRS for sport practice appears to represent an advancement yet there is a need to examine the validity of this catalog of items. As such, we sought to build on Bartulovic et al.'s practice-specific modifications, to explore the factor structure and criterion validity of their inventory relative to the full catalog of items (with commensurate practice-specific modifications) initially advanced in Toering et al.'s SRL-SRS.

Second, SRL assessment may need to capture greater breadth in conceptually relevant sub-processes. Although the SRL-SRS has loose conceptual basis in Zimmerman (1998, 2000) model and Ertmer and Newby's (1996) expert learner model, several SRL sub-processes (Winne and Hadwin, 1998; Zimmerman, 2000; see Pintrich, 2000) have been underrepresented in the extant SRL-SRS tools. In reviewing the prior SRL-SRS inventories relative to conceptual models, we contend that self-evaluation, adaptive inferences, and goal setting have not been assessed sufficiently. SRL-SRS items have focused on self-evaluation for the correctness of training, but have not assessed judgments relative to one's own standards or past performances (Zimmerman, 1998; e.g., Winne and Hadwin, 1998). Items have yet to ask athletes to evaluate progress in practice tasks compared to goals or previous task performance, performance across time, or feeling of movements. Self-reflection items have been devoid of content specifying the targets of reflection. Items have yet to assess how athletes judge reflection in regards to adjustment and setting of goals, and how reflection informs future planning (i.e., adaptive inferences; Zimmerman, 2000). Goal sub-processes are central to SRL models (Pintrich, 2000; Zimmerman, 2008), yet there remains a need to assess sub-processes associated with athletes' setting of specific goals, process and outcomes goals, effort goals, and for sub-processes by which athletes prioritize goals, as they relate to practice.

The SRL-SRS, modified specifically for sport practice, holds promise as a method for sport expertise research that assesses conditions for optimal practice (e.g., McCardle et al., 2017) and possibly Ericsson et al.'s (1993) notion of deliberate practice (Young and Baker, 2017). In pursuit of this, however, we contend that any future SRL-SRS catalog should more explicitly consider content to assess a key self-process of deliberate practice, *concentration*. Further, we posit that tests of criterion validity should examine a key tenet of any expert development framework, *skill group discrimination*.

Prior work on deliberate practice established concentration (i.e., sustained focus and mental effort toward a purposefully designed task) as a hallmark of optimal training conditions (Starkes et al., 1996; Young and Salmela, 2002). In previous SRL-SRS research, athletes' reports for *effort* distinguished between physical preparation activities and deliberate practice, with physical effort characterizing the former activities (Bartulovic et al., 2018). Bartulovic et al. (2018) acknowledged the need for a mental effort subscale, suggesting it may more likely characterize deliberate practice. We therefore aimed to test an added subscale that asks athletes to judge their proclivity to concentrate during various practice conditions. Altogether, our objective was to test the validity of an expanded catalog of SRL-SRS items that included concentration and added content for evaluating, reflecting, and goal setting, relative to Toering et al.'s and Bartulovic et al.'s inventories.

The establishment of validity necessitates tests for factorial validity (psychometrics and their relation to conceptually pertinent content) as well as criterion validity. With respect to the latter, many expertise development researchers hold that a phenomenon of interest (in our case, facets of SRL-SRS) should validly discriminate experts from less-experts (Ericsson and Smith, 1991; e.g., Abernethy et al., 1993) and, ideally, such discrimination should show correspondence across increasingly skilled groups. Baker and Young (2014) suggested SRL of sport training may be an individual difference variable that impacts athletes' practice and, thus, contributes to differences in acquired skill group status. Thus, we aimed to submit any psychometrically sound survey inventory to tests of criterion validity, to examine how SRL-SRS sub-processes distinguish multiple skill groups.

The overarching purpose was to examine the reliability and validity of an athlete self-report survey for SRL pertaining to sport practice. We addressed three specific aims. First, we aimed to confirm the factor structure of a practice-specific inventory comprised of items from Toering et al. and from Bartulovic et al. Second, we sought to explore the factor structure of an expanded inventory that included new content for four subscales, and to contrast the resulting model fit indices with the Toering et al. and the Bartulovic et al. version. These two objectives were pursued in Phase 1 of the study. Third, in Phase 2, we tested the group discrimination hypothesis to determine the criterion validity associated with resulting SRL inventories from Phase 1.

## Methods

### Participants and Procedure

Competitive athletes (*N* = 549) between 13 and 81 (*M* = 26.45, *SD* = 12.66; 55% female; *n* = 3 unspecified gender) years old were recruited via sport organizations. They competed as individual (64%) or team athletes (36%) in powerlifting (*n* = 119), volleyball (71), athletics (57), Olympic weightlifting (37), speed skating (34), swimming (32), basketball (22), curling (14), rugby (14), and 28 other sports (with 10 participants or fewer). Of the 549 respondents, those demonstrating survey fatigue (i.e., who failed to complete portions at the end of the survey; *n* = 50), and who were more than 2.5 *SD* above the age mean (*n* = 19) were removed from further analyses. The final sample comprised 482 athletes (*M age* = 25.3, *SD* = 10.62, range = 13-58 years), of which 12.2% had some missing data. Missing values analyses indicated data were missing at random; they were addressed using robust maximum likelihood estimation in Mplus.

We contacted sport organizations who forwarded an email containing a link to the survey to senior athletes (18 years and older) or parents of junior athletes (13–17 years old) within their organization. Once parents provided written informed consent, junior athletes were forwarded the link, where they provided informed assent. Senior athletes provided written informed consent. All measures were provided online in the *FluidSurveys* platform. Completion of the survey took approximately 20 min. The research ethics board of the leading institution approved all procedures.

### Survey Measures

Participants first completed items related to demographic and sport history information, reporting their main sport, number of years they had been training, and current weekly training hours. They reported their *highest performance level* ever, as a junior or as a senior, from five options (Hopwood, 2013): competing against athletes/teams (a) from neighborhoods across one's city (i.e., *local* level); (b) against athletes from different cities/towns in one's district (*regional*); (c) against athletes from different regions of one's province/state (*provincial*); (d) against athletes who represent different provinces/states (*national*); and (e) against those representing different countries (*international*). To enhance reliability, we instructed them to complete sport history and performance level information using external sources for recall: 66% acknowledged using one or more resources (e.g., personal training log, online archived results) to facilitate their responses.

#### Sport Practice Version of the Self-Regulated Learning—Self-Report Scale

Athletes responded to our survey inventory based on Bartulovic et al.'s (2017) sport practice version (SPV) of the SRL-SRS (see Table 1, section A). The SPV exhibits the same subscales as Toering et al.'s (2012a) SRL-SRS; however, unlike the original instrument, which was dispositional, the SPV had been preliminarily validated to ensure item specificity for sport practice. Of the 31 items on the SPV, we removed two from the effort subscale (“*I work as hard as possible on all tasks at practice*”; “*I work hard at practice on a task even if it is not important*”) because they were at odds with the deliberate practice framework (Ericsson et al., 1993) that focuses athletes' efforts on only the most relevant tasks. We modified one item in *planning* that was double-barreled (“*Before I do a practice task, I figure out my goals and what I need to do to achieve them*”) to derive items 20 and 21 in Table 1. We also reclaimed items from Toering et al.'s (2012a) work that had not been retained in Bartulovic et al.'s final SPV (see Table 1, section B). These items had conceptual relevance and ensured we had more than three items for all subscales. We phrased these reclaimed items specifically toward sport practice tasks. Altogether, we collated 35 SPV items that we refer to as the *initial items* or the *initial measurement model* (Table 1, sections A and B). Finally, we renamed the *self-efficacy* scale as *self-efficacy for challenges* (*SEC*) to reflect the nature of the items that address learners' confidence for facing difficulties, rather than their confidence to perform the tasks themselves (see Table 1 for item wording).

**Table 1:** Derivation of self-regulated learning items on each of the intended subscales for the initial and extended models.

**#**	**Item**	**Initial subscale^**a**^**	**Extended subscale^**b**^**
**SECTION A: BARTULOVIC ET AL. SPORT PRACTICE ITEMS BASED ON TOERING ET AL**.			
01	I determine how to approach a practice task before I begin.	Planning	Planning
02	Before practice tasks, I carefully plan my course of action.	Planning	Planning
05	I check how well I am doing during practice tasks.	Self-monitoring	Self-monitoring
06	I look back to check if what I did in practice was right.	Evaluating	Evaluating
10	I think about what a practice task requires me to do before I do it.	Planning	Planning
11	I clearly plan my course of action before starting practice tasks.	Planning	Planning
14	While I am engaged in a practice task, I know how much of it I still have to complete.	Self-monitoring	Self-monitoring
15	I look back to see if I did the correct procedures at practice.	Evaluating	Evaluating
19	Before I do a practice task, I think through the steps in my mind.	Planning	Planning
20	Before practice tasks, I figure out what I need to do to accomplish my goals.^c^	Planning	Planning
21	Before practice tasks, I figure out my goals.^c^	Planning	Goal setting
23	I double-check to make sure I did practice tasks right.	Evaluating	Evaluating
24	I check aspects of my workout while doing it.	Self-Monitoring	Self-monitoring
26	When thinking about my practice, I reflect about my strengths and weaknesses.	Reflecting	Reflecting
29	I develop a plan for resolving difficulties at practice.	Planning	Planning
32	I check my work all the way through a practice session.	Self-monitoring	Self-monitoring
33	After finishing, I look back on practice tasks to evaluate my performance.	Evaluating	Evaluating
35	I think about my past experiences at practice to gain new insights.	Reflecting	Reflecting
37	I know how to handle unforeseen situations during practice, because I am resourceful.	SEC	SEC
38	No matter what comes my way at practice, I am usually able to handle it.	SEC	SEC
39	Even when I don't like a task during practice, I work hard.	Effort	Effort
41	I concentrate fully when I do a task at practice.	Effort	Concentration
43	When facing difficulties at practice I can rely on my coping abilities.	SEC	SEC
44	I usually put forth my best effort when performing tasks at practice.	Effort	Effort
45	I am willing to do extra practice on tasks in order to acquire more skill.	Effort	Effort
49	I am confident that I can deal efficiently with unexpected events at practice.	SEC	SEC
50	I usually keep working hard even when sport training tasks become difficult.	Effort	Effort
51	If I'm not really good at a task, I can compensate by practicing hard.	Effort	Effort
55	When I am confronted with a difficulty during practice, I can usually find several solutions.	SEC	SEC
56	I don't give up at practice even if a task is hard.	Effort	Effort
**SECTION B: RECLAIMED ITEMS FROM TOERING ET AL**.			
03	I try to understand the goal of a practice task before I do it.	Planning	Planning
08	I reappraise my practice experiences so I can learn from them.	Reflecting	Reflecting
09	I reflect about how I can practice things better next time.	Reflecting	Reflecting
17	I reflect upon my actions at practice to see whether I can improve them.	Reflecting	Reflecting
28	Before practice tasks, I consider the parts of the task I have to complete.	Planning	Planning
**SECTION C: NEW ITEMS**			
04	I consciously have goals in mind for how hard I want to work at practice.	–	Goal setting
07	I compare my performance at practice with what I have done before.	–	Evaluating
12	I prioritize the most important goals I have for practice.	–	Goal setting
13	During practice, I consciously have goals in mind to improve how I train.	–	Goal setting
16	I evaluate whether I am getting better from practice to practice.	–	Evaluating
18	I think about my practice experiences so I can adjust my goals for practice.	–	Reflecting
22	I am aware of the outcomes I want to achieve during training	–	Goal setting
25	I compare my performance at practice with the goals that I have.	–	Evaluating
42	I reflect on my practice in order to set new goals.	–	Reflecting
30	I set specific training goals for myself.	–	Goal setting
31	I set personal training goals so I can check my progress.	–	Goal setting
34	I look back to judge if the way I practiced felt right.	–	Evaluating
36	I think about how practice has been going so I can plan for next time.	–	Reflecting
40	If I'm not really good at a task, I can compensate by fully concentrating.	–	Concentration
46	I do not lost my focus at practice, even if a task is hard.	–	Concentration
52	I usually block out distractors when performing sport training tasks.	–	Concentration
57	I usually stay focused even when tasks become difficult at practice.	–	Concentration
58	Even when I don't like a task during practice, I try to concentrate on what I'm doing.	–	Concentration

*A total of 53 SRL items; item numbers reflected order they were presented in the online survey with randomization on each survey page (1–18, 19–26, 37–48, 49–58) and included 4 items not submitted to analysis; SEC, self-efficacy for challenges*.

a *Refers to the intended subscale label from the sport practice version of the SRL-SRS (Bartulovic et al., 2017)*.

b Refers to our intended subscale with the extended list of items;

c *Split from Bartulovic/Toering item ‘Before I do a practice task, I figure out my goals and what I need to do to achieve them'*.

##### Extended item pool for under-represented subprocesses

Participants additionally responded to 18 new items we created: (a) 4 *evaluating* items assessing how one makes judgments in practice relative to one's own standards or past performance; (b) 3 *reflecting* items related to post-practice inferences about goals and planning; (c) 6 *goal setting* items assessing goal specificity, diversity, and prioritization of practice goals; and (d) 5 *concentration* items that assessed mental effort. Within this *extended measurement model* of 53 items, our plan was to append the new evaluating and reflecting items to existing subscales in the SPV, and to create two new subscales for *goal setting* (that included 1 *initial item* from planning and the 6 new items) and *concentration* (that included 1 *initial item* from effort and the five new items; see Table 1, section C).

Metacognitive items (planning, self-monitoring, evaluating, reflecting) were on a Likert scale anchored at 1 *never*, 4 *sometimes*, and 7 *always*; motivational items (SEC, effort, concentration) were on a scale from 1 *completely disagree* to 7 *completely agree*.

## Phase 1: Testing of Factor Structures

### Planned Analyses for the Initial Model

We conducted a confirmatory factor analysis (CFA) using robust maximum likelihood (MLR) estimation in Mplus (Muthén and Muthén, 2012). Model fit was assessed according to Hair et al. (2010) and Kline (2011): (a) non-significant chi-square; (b) root mean square error of approximation (RMSEA) < 0.08; (c) Tucker-Lewis Index (TLI) >0.90; and (d) comparative fit index (CFI) >0.90. We inspected modification indices > 1. For convergent validity of subscales, we computed average variance extracted (AVE) in items by the latent variable of the subscale, with values >0.5 indicating adequate convergence (Hair et al., 2010). We examined discriminant validity of subscales by calculating average shared variance (ASV) and maximum shared variance (MSV) among subscales, with values < 0.4 considered acceptable (Hair et al., 2010). We calculated composite reliability scores for each subscale, with values >0.7 indicating acceptable reliability (Hair et al., 2010).

### Results for the Initial Model

The CFA resulted in acceptable fit: χ^2^(545) = 1042.05, *p* < 0.001; RMSEA = 0.043 [0.039–0.047]; CFI = 0.923; TLI = 0.916. Item loadings were all >0.5, except for three items that remained >0.4 (see Table 2). Measures of convergent and discriminant validity as well as inter-factor correlations are in Table 3. Inter-scale correlations revealed that *evaluating* was very highly correlated with *reflecting* and with *self-monitoring*, a concern corroborated by an MSV of 1.00 for evaluating and self-monitoring. Further potential concerns were highlighted by modification indices related to items 29, 45, and 56, which evidenced cross-loadings on multiple subscales. As such, we elected to conduct refinements to the initial model to improve psychometrics.

**Table 2:** Factor loadings for the initial model: CFA results.

**Item**	**Factor loading**
**PLANNING**	
Before practice tasks, I figure out what I need to do to accomplish my goals. [20]	0.80
Before practice tasks, I figure out my goals. [21]	0.80
Before practice tasks, I carefully plan my course of action. [02]	0.76
I clearly plan my course of action before starting practice tasks. [11]	0.76
I think about what a practice task requires me to do before I do it. [10]	0.73
Before practice tasks, I consider the parts of the task I have to complete. [28]	0.72
I determine how to approach a practice task before I begin. [01]	0.69
Before I do a practice task, I think through the steps in my mind. [19]	0.68
I try to understand the goal of a practice task before I do it. [03]	0.60
I develop a plan for resolving difficulties at practice. [29]	0.58
**SELF-MONITORING**	
I check my work all the way through a practice session. [32]	0.72
I check aspects of my workout while doing it. [24]	0.68
I check how well I am doing during practice tasks. [05]	0.57
While I am engaged in a practice task, I know how much of it I still have to complete. [14]	0.41
**EVALUATING**	
I look back to see if I did the correct procedures at practice. [15]	0.71
After finishing, I look back on practice tasks to evaluate my performance. [33]	0.71
I look back to check if what I did in practice was right. [06]	0.70
I double-check to make sure I did practice tasks right. [23]	0.65
**REFLECTING**	
I reflect upon my actions at practice to see whether I can improve them. [17]	0.72
I think about my past experiences at practice to gain new insights. [35]	0.71
I reappraise my practice experiences so I can learn from them. [08]	0.69
I reflect about how I can practice things better next time. [09]	0.68
When thinking about my practice, I reflect about my strengths and weaknesses. [26]	0.59
**SELF-EFFICACY FOR CHALLENGES**	
I am confident that I can deal efficiently with unexpected events at practice. [49]	0.79
I know how to handle unforeseen situations during practice, because I am resourceful. [37]	0.77
No matter what comes my way at practice, I am usually able to handle it. [38]	0.75
When I am confronted with a difficulty during practice, I can usually find several solutions. [55]	0.74
When facing difficulties at practice I can rely on my coping abilities. [43]	0.72
**EFFORT**	
I usually keep working hard even when sport training tasks become difficult. [50]	0.83
I don't give up at practice even if a task is hard. [56]	0.80
I usually put forth my best effort when performing tasks at practice. [44]	0.72
Even when I don't like a task during practice, I work hard. [39]	0.71
I concentrate fully when I do a task at practice. [41]	0.65
I am willing to do extra practice on tasks in order to acquire more skill. [45]	0.49
If I'm not really good at a task, I can compensate by practicing hard. [51]	0.49

*Item numbers are indicated in block parentheses*.

**Table 3:** Factor reliability and validity and inter-factor correlations for initial, refined, and extended models.

					**Correlations**				
**INITIAL MODEL**									
**Factor**	**CR**	**AVE**	**MSV**	**ASV**	**PL**	**SM**	**EV**	**RE**	**SEC**
Planning	0.91	0.51	0.76	0.49					
Self-monitoring	0.68	0.31	1.00	0.59	0.87				
Evaluating	0.79	0.48	1.00	0.56	0.81	1.00			
Reflecting	0.81	0.46	0.85	0.55	0.84	0.87	0.92		
SEC	0.87	0.57	0.46	0.19	0.37	0.37	0.29	0.39	
Effort	0.85	0.47	0.46	0.27	0.42	0.49	0.45	0.52	0.68
					**Correlations**				
**REFINED MODEL**									
**Factor**	**CR**	**AVE**	**MSV**	**ASV**	**PL**	**CH**	**E/R**	**SEC**	
Planning	0.92	0.58	0.76	0.41					
Checking	0.81	0.58	0.74	0.41	0.70				
Evaluating/ Reflecting	0.81	0.51	0.76	0.51	0.87	0.86			
SEC	0.87	0.57	0.34	0.20	0.40	0.33	0.43		
Effort	0.88	0.60	0.36	0.30	0.46	0.53	0.60	0.59	
					**Correlations**				
**EXTENDED MODEL**									
**Factor**	**CR**	**AVE**	**MSV**	**ASV**	**PL**	**E/R**	**SEC**		
Planning	0.85	0.45	0.69	0.30					
Evaluating/ Reflecting	0.85	0.39	0.69	0.28	0.83				
SEC	0.87	0.57	0.69	0.27	0.32	0.25			
Effort/ Concentration	0.89	0.52	0.69	0.28	0.31	0.27	0.83		

*CR, composite reliability; AVE, average variance explained; MSV, maximum shared variance; ASV, average shared variance; PL, planning; SM, self-monitoring; EV, evaluating; RE, reflection; SEC, self-efficacy for challenges; CH, checking; E/R, evaluating/reflecting; all correlations significant at p < 0.05*.

#### Refinements to the Initial Model

We prioritized data-driven psychometric statistics, although we did consider conceptual interpretations of items on their latent factors. We adopted a two-stage approach using two randomly split samples; *subsample 1* (*n* = 241) for exploring factor structure and a hold-back *subsample 2* (*n* = 241) for validation purposes. *T*-tests on age, skill level, and amount of weekly practice indicated no differences between the subsamples, all *t*s < |0.8|, *p* > 0.47. Chi-square goodness-of-fit tests further indicated equivalent distributions of sex, individual/team, and junior/senior athletes, all χ^2^ <0.8, *p* > 0.40.

Using subsample 1, we conducted exploratory structural equation modeling (ESEM), a blend of EFA and CFA analytics (Marsh et al., 2014). In ESEM, the researcher can specify items to load on particular factors, but it also allows all items to load on all factors, thereby attenuating inter-factor correlations seen in CFA. Starting with all 35 items and the 6 specified subscales of the initial model, we conducted the ESEM using robust maximum likelihood and target rotation with one item per subscale to load at approximately 1. The majority of model fit indices were acceptable, though TLI was low: χ^2^ (400) = 648.01, *p* < 0.001; RMSEA = 0.051 [0.043–0.058]; CFI = 0.918; TLI = 0.878. Moreover, two issues came to light: (a) none of the *self-monitoring* items loaded >0.4, with values ranging from −0.1 to 0.25; and (b) there were several items cross-loading between *evaluating* and *reflecting*. This suggested *self-monitoring, evaluating*, and *reflecting* subscales were not psychometrically distinct. Due to the high number of estimated parameters, we decided to be cautious in interpreting results and opted to restart refinements with a more conventional EFA approach.

Thus, we ran a series of EFAs (MLR estimation, geomin rotation) on subsample 1, iteratively identifying problematic items, removing them from the model, and re-running the EFA (see Appendix A in Supplementary Material for the rationale and statistics underlying item removal); when we removed an item, we were confident that it did not share conceptual similarities with remaining items on the same factor. The resultant 26-item model had 5 identifiable factors: *planning, checking, reflecting, SEC*, and *effort* (Table 4). We retained two items with factor loadings < 0.4 because they had very good conceptual fit with the rest of their subscale; in the case of *checking*, removing the item would have left only two items.

**Table 4:** Factor matrices for the refined model: EFA and CFA results.

	**EFA Factors**					
**Item**	**1**	**2**	**3**	**4**	**5**	**CFA**
**PLANNING**						
I determine how to approach a practice task before I begin. [01]	**0.77**	0.04	−0.20	−0.07	0.04	0.76
Before practice tasks, I figure out my goals. [21]	**0.72**	−0.06	0.20	0.03	−0.03	0.82
Before practice tasks, I carefully plan my course of action. [02]	**0.69**	0.26	−0.14	0.05	−0.07	0.78
Before practice tasks, I figure out what I need to do to accomplish my goals. [20]	**0.63**	−0.11	0.29	0.01	−0.01	0.85
Before practice tasks, I consider the parts of the task I have to complete. [28]	**0.59**	−0.03	0.17	0.00	0.14	0.72
I clearly plan my course of action before starting practice tasks. [11]	**0.56**	0.20	0.05	0.21	−0.13	0.79
I think about what a practice task requires me to do before I do it. [10]	**0.55**	0.10	0.11	−0.05	0.15	0.75
I try to understand the goal of a practice task before I do it. [03]	**0.55**	−0.00	−0.10	0.13	0.14	0.67
Before I do a practice task, I think through the steps in my mind. [19]	**0.51**	0.03	0.21	−0.05	0.06	0.69
**CHECKING**						
I look back to check if what I did in practice was right. [06]	−0.01	**0.91**	−0.01	0.06	0.00	0.74
I look back to see if I did the correct procedures at practice. [15]	0.21	**0.46**	0.24	−0.03	0.02	0.81
I check aspects of my workout while doing it. [24]	0.27	**0.39**	0.12	−0.17	0.15	0.74
**EVALUATING/REFLECTING**						
I reflect upon my actions at practice to see whether I can improve them. [17]	−0.01	0.05	**0.64**	0.01	0.14	0.78
After finishing, I look back on practice tasks to evaluate my performance. [33]	0.08	0.18	**0.58**	−0.01	−0.02	0.72
I think about my past experiences at practice to gain new insights. [35]	0.26	0.11	**0.47**	0.05	−0.08	0.71
When thinking about my practice, I reflect about my strengths and weaknesses. [26]	0.10	0.03	**0.37**	0.06	0.07	0.64
**SELF–EFFICACY FOR CHALLENGES**						
I know how to handle unforeseen situations during practice, because I am resourceful. [37]	0.00	0.03	0.05	**0.78**	−0.00	0.78
When I am confronted with a difficulty during practice, I can usually find several solutions. [55]	−0.01	−0.01	0.11	**0.75**	0.03	0.71
I am confident that I can deal efficiently with unexpected events at practice. [49]	0.02	0.01	−0.05	**0.64**	0.26	0.78
When facing difficulties at practice I can rely on my coping abilities. [43]	0.10	0.07	−0.01	**0.58**	0.14	0.73
No matter what comes my way at practice, I am usually able to handle it. [38]	0.01	−0.10	0.01	**0.57**	0.24	0.78
**EFFORT**						
I usually keep working hard even when sport training tasks become difficult. [50]	0.08	−0.05	−0.02	−0.01	**0.84**	0.87
I don't give up at practice even if a task is hard. [56]	−0.07	−0.01	−0.01	0.31	**0.61**	0.80
I usually put forth my best effort when performing tasks at practice. [44]	−0.10	0.10	0.13	0.10	**0.59**	0.73
Even when I don't like a task during practice, I work hard. [39]	0.07	0.03	−0.05	0.06	**0.57**	0.81
I concentrate fully when I do a task at practice. [41]	0.03	0.01	0.19	0.17	**0.50**	0.64

*Item numbers are indicated in block parentheses. Bold values indicate factor loadings for items that constitute the factor*.

Finally, we conducted a CFA on this refined model using subsample 2. Fit indices were acceptable, χ^2^ (289) = 538.48, *p* < 0.001; RMSEA = 0.060 [0.052–0.068]; CFI = 0.915; TLI = 0.905, and item loadings were all >0.63 (Table 4). Measures of convergent and discriminant validity as well as inter-factor correlations are presented in Table 3; composite reliability and convergent validity were acceptable and discriminant validity was improved over the initial model.

### Planned Analyses for the Extended Model

We took a two-stage approach beginning with a series of EFAs, with the extended catalog of 53 items on subsample 2, and followed with a validation process using CFA with the remaining hold back subsample 1. With subsample 2, we ran iterative EFAs in Mplus using MLR estimation and geomin rotation, an oblique rotation that allows for correlations between factors. In Mplus, results are provided for models with 1 to *n* factors; in each iteration, we chose the model for further inspection based on best model fit using the aforementioned criteria as well as Eigenvalues. For the model with best fit, we then inspected factor pattern and factor structure coefficients (Russell, 2002). Items were considered for removal if they demonstrated low factor loadings (< 0.4), low within-factor correlations (< 0.5), and/or values that < |0.2| difference between the primary loading and any cross-loading value. We also considered conceptual fit with other items in a factor. After the iterative EFAs and exclusion of ill-fitting items, we planned to settle on a final extended model and to submit this model to a CFA using subsample 1.

### Results for the Extended Model

Our initial EFAs highlighted problems associated with the new *goal setting* subscale. Seventeen items (nearly one third of the inventory) clustered together on a factor that focused on goals. This may have been due to the fact many of the new items we created for other subscales also drew on goal subprocesses (e.g., “*I reflect on my practice in order to set new goals*”, a *reflecting* item). Thus, this 17-item factor included *planning, evaluating*, and *reflecting* items in addition to the newly designed *goal setting* items and was conceptually difficult to interpret in relation to other factors that also addressed evaluating and reflecting but did not include goals. Due to the fact that goal setting distorted all the other subscales, we removed all 6 of our new goal-setting items and re-began the analysis with 47 items.

A series of EFAs resulted in the removal of 15 items (see Appendix B in Supplementary Material for the rationale and statistics underlying item removal). After the first four iterations, there was a coherent factor we labeled *checking*, similar to that in the refined model. This was surprising as we anticipated these items related to checking correctness of practice might load with *evaluating* given the added evaluating items. We were also concerned with items that had low loadings (items 06 and 15 loaded >0.4; items 03 and 32 loaded >0.3) as well as cross-loading on other factors. Thus, we opted to remove the highest loading item (item 15) from *checking*. In two further EFA iterations, the other two items that loaded most highly on checking were problematic and were removed (items 06 and 32), while the lone remaining checking item (item 03) loaded on *planning*. Although we intended for effort and concentration items to load on separate subscales, these items continually loaded together. At this final point, the *extended model* included 29 items across 4 factors (see Table 5): *planning, evaluating/reflecting, effort/concentration*, and *SEC*. A CFA on subsample 1 revealed the model had acceptable fit: χ^2^ (371) = 561.89, *p* < 0.001; RMSEA = 0.046 [0.038–0.054]; CFI = 0.926; TLI = 0.919. Item loadings were >0.5 with two exceptions. Table 3 shows that composite reliability was acceptable; convergent validity was acceptable for *effort/concentration* and *SEC*, but AVE was low (< 0.5) for *planning* and *evaluating/reflecting*. Discriminant validity values were acceptable for ASV but high for MSV (>0.4).

**Table 5:** Factor matrices for the extended model: EFA and CFA results.

	**EFA Factors**				
**Item**	**1**	**2**	**3**	**4**	**CFA**
**PLANNING**					
Before practice tasks, I carefully plan my course of action. [02]	**0.88**	−0.04	−0.11	0.08	0.75
I clearly plan my course of action before starting practice tasks. [11]	**0.81**	0.09	−0.18	0.07	0.74
I determine how to approach a practice task before I begin. [01]	**0.75**	−0.00	0.03	0.04	0.61
Before practice tasks, I figure out what I need to do to accomplish my goals. [20]	**0.66**	0.21	0.08	−0.07	0.73
I think about what a practice task requires me to do before I do it. [10]	**0.56**	0.21	0.10	−0.04	0.68
I try to understand the goal of a practice task before I do it. [03]	**0.54**	0.11	0.11	−0.02	0.54
Before I do a practice task, I think through the steps in my mind. [19]	**0.47**	0.19	0.10	0.05	0.65
**EVALUATING/REFLECTING**					
I think about my past experiences at practice to gain new insights. [35]	0.04	**0.77**	−0.12	0.21	0.74
I consider how practice has been going so I can plan for next time. [36]	0.10	**0.75**	−0.11	0.11	0.64
I look back to judge if the way I practiced felt right. [34]	−0.08	**0.71**	0.05	−0.01	0.55
I evaluate whether I am getting better from practice to practice. [16]	0.05	**0.66**	0.08	−0.04	0.62
When thinking about my practice, I reflect about my strengths and weaknesses. [26]	−0.03	**0.64**	0.09	0.11	0.49
I reflect on my practice in order to set new goals. [27]	0.20	**0.62**	0.03	−0.02	0.71
I think about my practice experiences so I can adjust my goals for practice. [18]	0.22	**0.58**	0.03	−0.01	0.73
I compare my performance at practice with what I have done before. [07]	0.14	**0.55**	0.00	−0.08	0.39
I compare my performance at practice with the goals that I have. [25]	0.28	**0.50**	0.02	−0.12	0.69
**EFFORT/CONCENTRATION**					
Even when I don't like a task during practice, I work hard. [39]	0.03	0.02	**0.85**	−0.10	0.59
I usually keep working hard even when sport training tasks become difficult. [50]	0.04	0.02	**0.84**	0.01	0.73
I don't give up at practice even if a task is hard. [56]	0.05	−0.07	**0.79**	0.06	0.77
Even when I don't like a task during practice, I try to concentrate on what I'm doing. [58]	0.13	−0.08	**0.71**	0.01	0.77
I usually stay focused even when tasks become difficult at practice. [57]	0	0.08	**0.62**	0.19	0.82
I usually put forth my best effort when performing tasks at practice. [44]	−0.05	0.23	**0.60**	0.04	0.68
I concentrate fully when I do a task at practice. [41]	−0.06	0.28	**0.46**	0.13	0.70
I do not lose my focus at practice, even if a task is hard. [46]	0.14	0.05	**0.43**	0.14	0.66
**SELF–EFFICACY FOR CHALLENGES**					
No matter what comes my way at practice, I am usually able to handle it. [38]	−0.04	0.11	0.06	**0.75**	0.73
I know how to handle unforeseen situations during practice, because I am resourceful. [37]	0.08	0.05	0.01	**0.73**	0.76
When facing difficulties at practice I can rely on my coping abilities. [43]	0.13	−0.06	−0.02	**0.70**	0.71
I am confident that I can deal efficiently with unexpected events at practice. [49]	−0.06	0.03	0.22	**0.66**	0.80
When I am confronted with a difficulty during practice, I can usually find several solutions. [55]	0.17	−0.11	0.07	**0.63**	0.773

*Item numbers are indicated in block parentheses. Bold values indicate factor loadings for items that constitute the factor*.

### Phase 1 Discussion

In Phase 1, we aimed to analyze the best fitting factor structure for assessing SRL in a sport practice setting. Our goal was to assess the psychometrics of the *initial model* (SPV, including some reclaimed items from prior SRL work in sport) and an e*xtended model* that included new items to broaden the conceptual breadth of the SPV. During our analyses, we also explored a refined version of the initial model, which we labeled the *refined model*.

#### What Have we Learned About the Psychometrics for Self-report of Self-regulated Learning?

Although one aim was to extend the conceptual breadth of the SRL-SRS, as we went from the initial, to the refined, to the extended model, we ironically decreased the number of subscales and, thus, the number of SRL concepts represented. Based on observations during our iterative testing, we identified three particular areas for discussion.

##### The challenge of capturing goal subprocesses

Our EFAs with the extended model underscored the centrality of goals, as the inclusion of goals brought all other metacognitive subprocesses together into one subscale; goals converged with planning, self-monitoring, evaluating, and reflecting. Indeed, our extensions of the evaluating and reflecting subscales included items related to comparison to, and adaptation of, athletes' goals. The question becomes whether it is important to have a distinct measure of goal setting or it is safe to assume that goals are inherent within other SRL subprocesses? In applied settings, knowledge around athletes' goal setting tendencies might be useful; however, given the ubiquity of strategic goal setting subprocesses, their assessment may not reveal much variation across individuals. Further consideration of the centrality and measurement of goal setting is needed.

##### Convergence of self-monitoring, evaluating, and reflecting

Toering et al. (2011, 2012b) development of the SRL-SRS, there were separate subscales for each of self-monitoring, evaluating, and reflecting. Although we sought to maintain distinctiveness of these subscales, they became the main source of multicollinearity in our initial model, and we saw less clarity between these subprocesses in both our refined and extended models. Conceptualizations of SRL suggest that such multi-collinearity should be expected. According to Zimmerman (2000), self-monitoring and evaluating both involve a learner's comparison of current performance against a standard, though self-monitoring is located in the performance phase (comparisons occurring *during* learning activities) and evaluating is located in the self-reflection phase (comparisons occurring *after* learning activities). Zimmerman also conceptualizes reflection as a phase in his model, comprised of self-evaluating subprocesses and other post-learning activity evaluations related to attributions, self-satisfaction, and adaptive inferences. Traditionally, self-monitoring has been conceptualized (Spates and Kanfer, 1977) as comprising the subprocess of checking the current state of one's progress and also self-evaluating what has been checked against a standard. Thus, there is conceptual overlap between many SRL subprocesses; they are intertwined and enacted proximally to one another. It may be unrealistic to develop an athlete survey tool, especially one that is completed outside the immediate practice situation, which separates these subprocesses as distinct measureable factors at a level that satisfies strict psychometric criteria.

##### Keeping effort and concentration distinct

Highly relevant forms of deliberate practice can be distinguished by physical effort and mental concentration; however, many highly important activities associate more strongly with a demand for mental effort and focus, than physical demands (e.g., Young and Salmela, 2002). Thus, we explored whether we could develop novel items for concentration in our extended model that would remain distinct from Toering et al.'s physical effort subscale. This was not achieved. The concentration and physical effort items may have converged together because the concentration items were phrased in parallel with the effort items or because the “effort” items did not refer explicitly enough to physical effort. Their convergence may also be attributed to the fact these items do not represent distinct enough concepts to warrant separate subscales. Most sport tasks arguably necessitate both effort and concentration and although there may be varying degrees assigned to each (depending on the sport and task), the grain of measurement in a self-report survey may be too large to maintain the psychometric distinctiveness for such ratings. Perhaps the fact that engaging metacognitive subprocesses is effortful to begin with means measuring effort as a subscale is unessential to an SRL self-report measure.

#### Which Model Has the Best Factor Structure?

Table 6 (top section) compares the models, displaying key psychometric indices. Although we hoped one model would emerge as a better representation of the data, this did not unfold conclusively: CFA model fit was similar across all models. It was promising that we saw acceptable model fit for our refined and extended models given the strict parameters imposed by CFA (Marsh et al., 2014), providing evidence for factor validity. Overall, subscales consistently demonstrated acceptable levels of internal reliability. At the very least, we believe that the sport-specific wording of the items shared by all three models is a step forward in developing the most valid and reliable version of an SRL survey. Unlike Toering et al. (2012b, 2011) formative work on the development of the SRL-SRS, where they employed a dispositional instrument that retained phrasing suited to mathematics scholarship and in-class computational desk tasks by students, the revision of SPV items allowed participants to report what they do for their sport training.

**Table 6:** Comparisons between the measurement models based on factorial validity indices and criterion validity analyses.

	**Initial**	**Refined**	**Extended**
**SCALE**			
Items	35	26	29
Factors	6	5	4
**CFA MODEL FIT**			
CFI	0.923	0.915	0.926
TLI	0.916	0.905	0.919
RMSEA	0.053 [0.039–0.047]	0.060 [0.052–0.068]	0.046 [0.038–0.054]
**CONVERGENT AND DIVERGENT VALIDITY**			
AVE	0.31–0.057	0.51–0.60	0.39–0.57
MSV	0.46–1.00	0.34–0.76	0.69–0.69
ASV	0.19–0.59	0.20–0.51	0.27–0.030
**CRITERION VALIDITY**			
*pη^2^*	0.038	0.029	0.023
Effect size	Small–medium	Small–medium	Small–medium
Metacognitive subscales with significant differences (*Pη^2^*)	Reflecting (0.035)	Evaluating/Reflecting (0.032)	–
Motivational subscales with significant differences (*Pη^2^*)	Effort (0.038) SEC (0.053)	Effort (0.043) SEC (0.051)	Effort/Concentration (0.043) SEC (0.049)

*Criterion validity effect sizes are for senior athletes only; pη^2^, partial eta^2^ values; SEC, self–efficacy for challenges; AVE, average variance explained, values >0.5 indicate adequate convergence; MSV, maximum shared variance, and ASV, average shared variance, values < 0.4 indicate adequate divergence*.

There was some psychometric evidence that concerned us with respect to the *initial model*. For example, statistics related to discriminant validity of the subscales revealed issues regarding the overlap between self-monitoring, evaluating, and reflecting. The interfactor correlations were high, with two subscales correlating >0.9 and four subscales correlating >0.8. Although Toering et al. (2012a) examined students who were not necessarily athletes, their inter-scale correlations in the examination of the original SRL-SRS work ranged from 0.34 to 0.63. The interfactor multicollinearity we found for our initial model was congruent with Bartulovic et al.'s (2017) values obtained with the SPV among athletes, where subscale correlations ranges were quite high, up to 0.95. Overall, however, we felt we were unable based on Phase 1 alone to recommend a model for other researchers.

## Phase 2: Testing Skill Group Differences for Criterion Validity

The purpose was to examine initial evidence of criterion validity in the hope that one model would emerge more conclusively as a candidate for further use. Hopwood and Donnellan (2010) questioned whether questionnaire development should predominantly rely on psychometric analysis, as has been the trend, suggesting other forms of validity be considered to supplement factorial validity. Our approach in Phase 2 was aligned with this notion. In keeping with a major tenet of any expert development approach in sport (Ericsson and Smith, 1991; e.g., Abernethy et al., 1993; Tedesqui and Young, 2017), we tested the *group discrimination proposition*; i.e., the extent to which subscales scores from each model could discriminate between escalating performance groups.

### Planned Analyses

We compared the relationships between scores for the three models and our criterion of performance level. We used athletes' highest reported performance level: *local, regional, provincial, national*, or *international* and analyzed junior (*n* = 142) and senior athletes (*n* = 369) separately (32 athletes who reported both highest junior and senior levels due to their age being close to the senior cutoff age of 18, were included in both analyses). The smallest cells of respondents pertained to the least skilled groups. Thus, we combined *local* and *regional* athletes into one group (referred to as *local/regional*).

For juniors, we conducted separate analyses of variance (ANOVA) for group differences for each of the *initial, refined*, and *extended* subscales. For seniors, we conducted multivariate analyses of variance (MANOVAs) on subscales scores as a function of 4 performance groups, for each model. We followed up significant omnibus MANOVA tests with separate ANOVAs and least significant difference comparisons. Effect sizes were determined as partial eta^2^ values interpreted as 0.01 small 0.06 medium, and 0.14 large. We used direct discriminant analysis (DA) to follow-up on significant MANOVA effects, to examine the multivariate combination of SRL subprocesses that best distinguished groups (Field, 2005). We considered only discriminant functions that were significant and used a canonical correlation of 0.33 for considering a substantial contribution from any subscale to the function (Tabachnick and Fidell, 2013).

### Results

**Figure 1:**
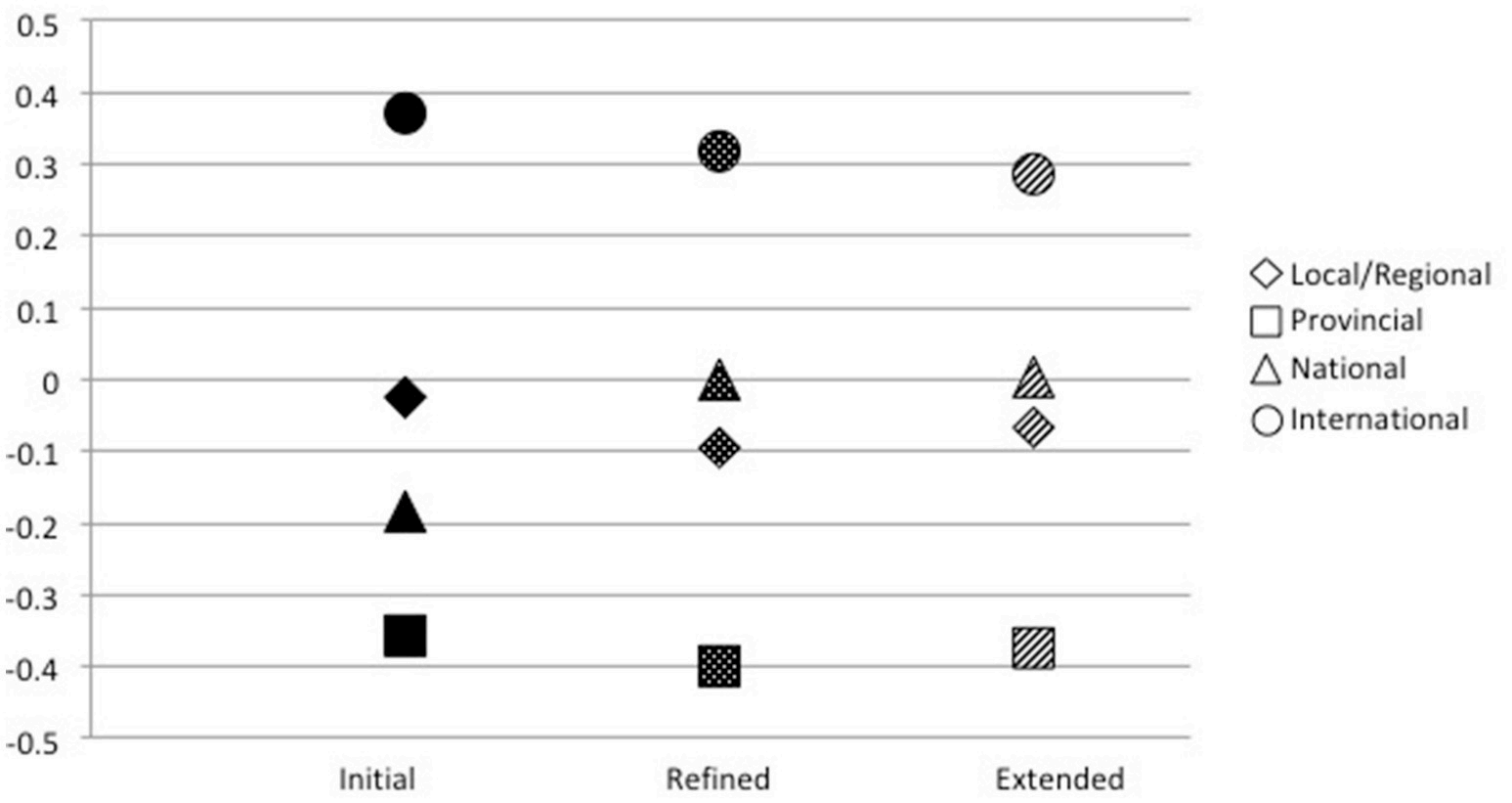
Discriminant analysis function 1 scores and standardized canonical coefficients in order of highest to lowest structure loadings.

Descriptive statistics for all subscales by performance levels are found in Table 7. We describe results for each measurement model separately, but a comparison of MANOVA effect sizes, ANOVA findings, and DA structure matrix for all models is displayed in Table 8 and comparison of DA function 1 scores are found in Figure 1.

**Table 7:** Means (standard deviations) for each subscale by each performance level group according to each measurement model.

	**Local/Regional**	**Provincial**	**National**	**International**
**JUNIOR ATHLETES**				
**Initial model**			
Planning	4.71 (1.13)	5.16 (1.27)	5.08 (1.13)	5.25 (1.19)
Self–monitoring	5.57 (0.73)	5.55 (1.15)	5.39 (0.95)	5.63 (0.81)
Evaluating	5.15 (0.95)	5.56 (1.16)	5.25 (1.11)	5.61 (0.95)
Reflecting	5.27 (0.82)	5.63 (1.02)	5.59 (1.00)	5.81 (0.86)
Effort	6.34 (0.59)	6.26 (0.58)	6.25 (0.56)	6.47 (0.41)
SEC	5.51 (0.83)	5.90 (0.65)	5.72 (1.01)	5.91 (0.71)
**Refined model**			
Planning	4.67 (1.16)	5.18 (1.30)	5.09 (1.15)	5.25 (1.17)
Checking	5.31 (0.85)	5.58 (1.20)	5.21 (1.12)	5.36 (1.05)
Evaluating/Reflecting	5.12 (1.01)	5.52 (1.21)	5.57 (0.97)	5.82 (0.79)
Effort	6.39 (0.68)	6.29 (0.63)	6.29 (0.57)	6.37 (0.50)
SEC	5.51 (0.83)	5.90 (0.65)	5.72 (1.01)	5.91 (0.71)
**Extended model**			
Planning	4.70 (1.09)	5.21 (1.29)	5.09 (1.16)	5.29 (1.16)
Evaluating/Reflecting	5.20 (0.91)	5.49 (1.19)	5.67 (0.94)	5.87 (0.79)
Effort/Concentration	6.26 (0.66)	6.17 (0.72)	6.18 (0.59)	6.31 (0.55)
SEC	5.51 (0.83)	5.90 (0.65)	5.72 (1.01)	5.91 (0.71)
**SENIOR ATHLETES**			
**Initial model**			
Planning	5.43 (1.12)	5.32 (0.93)	5.32 (1.05)	5.61 (0.96)
Self–Monitoring	5.53 (1.17)	5.46 (1.00)	5.62 (0.87)	5.66 (0.93)
Evaluating	5.55 (1.21)	5.43 (1.06)	5.39 (1.07)	5.72 (1.01)
Reflecting	5.52 (1.16)	5.49 (0.89)	5.56 (1.00)	5.90 (0.85)
Effort	6.08 (0.73)	5.88 (0.64)	6.03 (0.68)	6.20 (0.59)
SEC	5.66 (0.96)	5.37 (0.82)	5.61 (0.89)	5.87 (0.75)
**Refined model**			
Planning	5.46 (1.15)	5.35 (0.94)	5.36 (1.07)	5.64 (0.97)
Checking	5.41 (1.14)	5.22 (1.27)	5.25 (1.18)	5.58 (1.15)
Evaluating/Reflecting	5.55 (1.20)	5.51 (0.90)	5.66 (0.97)	5.92 (0.88)
Effort	6.15 (0.76)	5.91 (0.69)	6.12 (0.71)	6.28 (0.62)
SEC	5.66 (0.96)	5.37 (0.82)	5.61 (0.89)	5.87 (0.75)
**Extended model**			
Planning	5.45 (1.14)	5.37 (0.95)	5.38 (1.09)	5.67 (0.98)
Evaluating/Reflecting	5.66 (1.09)	5.60 (0.83)	5.70 (0.90)	5.85 (0.84)
Effort/Concentration	5.99 (0.82)	5.75 (0.73)	6.00 (0.68)	6.14 (0.65)
SEC	5.66 (0.96)	5.37 (0.82)	5.61 (0.89)	5.87 (0.75)

*All subscales on 7–point Likert scale; SEC, self–efficacy for challenges*.

#### Senior Athletes

##### Initial model subscales

The MANOVA indicated significant skill level differences: *Wilk's* λ = 0.89, *F*_(18, 939)_ = 2.16, *p* = 0.003, *partial* η^2^ = 0.038. There was a significant group difference for *reflecting, effort*, and *SEC, F*s > 4.09, *p*s < 0.005 (Table 8). ANOVAs for planning, self-monitoring, and evaluating were non-significant, *F*s < 2.0, *p*s > 0.10. Of three extracted discriminant functions, the first function accounted for 71.3% of between-group variance and 7.8% of the total relation between predictors and groups. After removal of the first function, the second (*p* = 0.33) and third functions (*p* = 0.56) were non-significant. The first function discriminated between the international athletes and the rest, with the largest difference between international and provincial athletes. The structure matrix (Table 8) indicated *SEC* and *effort* were the best variables for distinguishing international athletes from the rest; however, all variables except self-monitoring were above 0.4. *Self-monitoring* had a negative coefficient, suggesting less skilled groups reported more highly on self-monitoring but given that it had a relatively small loading (0.22), this variable had less of a role.

**Table 8:** Indices relating to criterion validity comparisons across the three measurement models.

**Initial**			**Refined**			**Extended**		
	***p***	***pη***^**2**^		***p***	***pη***^**2**^		***p***	***pη***^**2**^
**MANOVA**								
Full model		0.038	Full model		0.029	Full model		0.023
Planning	*ns*		Planning	*ns*		Planning	*ns*	
Self-monitoring	*ns*		Checking	*ns*		Evaluating/Reflecting	*ns*	
Evaluating	*ns*		**Evaluating/Reflecting**	**0.011**	**0.032**	**Effort/Concentration**	**0.002**	**0.043**
**Reflecting**	**0.007**	**0.035**	I > P	0.003		I > P	< 0.001	
I > N	0.011		I > LR	0.017		N > P	0.018	
I > P	0.003		**Effort**	**0.002**	**0.043**	**SEC**	**0.001**	**0.049**
I > LR	0.002		I > P	< 0.001		I > N	0.034	
**Effort**	**0.004**	**0.038**	N > P	0.032		I > P	<0.001	
I > N	0.047		**SEC**	**< 0.001**	**0.051**	N > P	0.039	
I > P	< 0.001		I > N	0.034				
**SEC**	**< 0.001**	**0.053**	I > P	< 0.001				
I > N	0.023		N > P	0.028				
I > P	< 0.001							
N > P	0.041							
LR > P	0.044							
**DISCRIMINANT ANALYSIS**								
	***CC***	***SM***		***CC***	***SM***		***CC***	***SM***
Planning	−0.04	0.44	Planning	−0.27	0.37	Planning	−0.05	0.41
Self-monitoring	−0.57	0.22	Checking	0.04	0.39			
Evaluating	0.26	0.43	Evaluating/Reflecting	0.44	0.63	Evaluating/Reflecting	0.08	0.41
Reflecting	0.55	0.63						
Effort	0.26	0.67	Effort	0.38	0.77	Effort/Concentration	0.47	0.84
SEC	0.65	0.79	SEC	0.62	0.84	SEC	0.64	0.91

*pη^2^ = partial eta^2^ values; canonical coefficients (CC) and structure matrix (SM) are only shown for the first discriminant function of each analysis; SEC, self-efficacy for challenges; LR, local/regional; P, provincial; N, national; I, international; ns, non-significant at p < 0.05. Bold refers to subscales with significant (p < 0.05) ANOVAs*.

##### Refined model subscales

The MANOVA revealed a significant skill level difference: *Wilk's* λ = 0.91, *F*_(15, 947)_ = 2.07, *p* = 0.009, *partial* η^2^ = 0.029. There were significant group differences for *evaluating/reflecting, effort*, and *SEC, F*s > 3.75, *p*s < 0.012 (see Table 8). ANOVAs for planning and checking were non-significant, *F*s < 1.9, *p*s > 0.14. Three functions were extracted; the first accounted for 81.8% of the between-group variance and 7.0% of the total relation between predictors and groups. After removal of the first function, the second (*p* = 0.64) and third functions (*p* = 0.57) were non-significant. The first function discriminated the international athletes from the rest, with the largest difference between international and provincial athletes. The structure matrix (Table 8) indicated *SEC* and *effort* best distinguished international athletes from the rest, with *evaluating/reflecting* also contributing strongly. *Planning* and *checking* did contribute to a lesser extent, but the planning coefficient was negative suggesting international athletes engage in less planning than the other groups.

##### Extended model subscales

The MANOVA indicated a group difference: *Wilk's* λ = 0.93, *F*_(12, 902)_ = 1.99, *p* = 0.022, *partial* η^2^ = 0.023. Significant group differences were evident for *effort/concentration* and *SEC, F*s > 5.13, *p*s < 0.003 (see Table 8). ANOVAs for planning and evaluating/reflecting were non-significant: *F*s < 1.7, *p*s >0.17. Of three functions extracted, the first function accounted for 88.1% of the between-group variance and 5.9% of the total relation between predictors and groups. After removal of the first function, the second (*p* = 0.82) and third functions (*p* = 0.89) were non-significant. The first function discriminated international athletes from the rest, with the largest difference between international and provincial athletes. The structure matrix (Table 8) showed *SEC* and *effort* were the best subscales for distinguishing international athletes, with *planning* and *evaluating/reflecting* also contributing.

#### Junior Athletes

None of the ANOVAs conducted for junior athletes for the subscales for initial, refined, or extended models were significant, all *F*s < 1.6, *p*s >0.24.

### Phase 2 Discussion

In Phase 2, we examined initial evidence of criterion validity comparing subscale scores across performance level groups, using best-ever achieved performance level as the criterion measure for validation, similar to other research that has used domain-general (Toering et al., 2009) and sport-specific SRL survey measures (Bartulovic et al., 2017). Subscale scores from each of the three candidate models were submitted to analyses.

Across all three measurement models, our results indicated international athletes use a combination of motivation (i.e., effort and SEC) and metacognition (i.e., checking and evaluating/reflecting) more than other athletes. Significant differences were found most often between international athletes and the national, provincial, and local/regional athletes, while few differences were found among these lower level groups. The DA results suggest international-level athletes are a unique group, aligning with prior research that suggests they have a SRL advantage compared to sub-elite athletes (Toering et al., 2009, 2012b; Jonker et al., 2010).

In each model, multiple subscales loaded significantly on the discriminant factor suggesting that a combination of many SRL subprocesses distinguishes international athletes. This is consistent with prior results (Bartulovic et al., 2017) in which a composite SRL score (i.e., the average of six subscales) more effectively distinguished elite athletes from both less-elite and recreational-competitive than constituent subprocesses (i.e., self-monitoring, planning, SEC, effort). In terms of explaining elite athletes' skill status, Bartulovic et al. (2017) acknowledged the contribution of constituent subprocesses was difficult to detect in simultaneous regressions but inferred that multiple subprocesses were synergistically contributing to the elite athletes' SRL advantage. Our results suggest similar synergies between subprocesses, though negative canonical coefficients relating to planning were counter-intuitive as they suggested international athletes were less likely to engage in planning. The structure matrix loadings for planning were generally low, suggesting its diminished role in SRL in our sample. It is possible our international athletes relied more heavily on their evaluating and reflecting processes to direct their self-regulatory engagement.

In summary, we found preliminary evidence for criterion validity in that subscale scores distinguished international athletes from the rest. A strong conclusion on criterion validity rests on future research showing more consistent discrimination between each escalating skill group. Across all models, our results highlighted motivational aspects of SRL (i.e., SEC and effort) as critically distinguishing multiple groups, which builds upon prior SRL self-report research. Among youth soccer players, Toering et al. (2009) reported differences between elite and sub-elite players for effort but not self-efficacy. In a multisport sample, Bartulovic et al. (2017) found effort and self-efficacy each discriminated three groups, with a corresponding advantage favoring increasing skill groups. Although Jonker et al. (2010) found no differences on self-efficacy or effort between elite and sub-elite athletes across various team and individual sports, expert basketball athletes reported higher self-efficacy than non-experts, whose self-efficacy was higher than novices based on micro-analytic *in situ* (during practice) methods (Cleary and Zimmerman, 2001). Although our findings highlighted a greater contribution by motivational relative to metacognitive subprocesses, further work is needed to better understand the interplay of motivation and metacognitive elements toward skill group status.

## General Discussion

We aimed to assess the factorial and criterion validity of the revised SPV and an extended version. The *initial* version was based on Toering et al.'s (2012a) SRL-SRS with modifications to the item wording and factor structure inherited from Bartulovic et al.'s (2017) SPV. In the initial model, we maintained the same factor structure as both Toering et al. and Bartulovic et al. Due to issues with multicollinearity identified in the initial model, we developed an additional, unplanned model, we labeled *refined*, in which we removed items and adjusted factors to create a more psychometrically sound model. In the *extended* version, we added items relating to concepts that we identified as being theoretically important but underrepresented in the SPV including goal setting, mental concentration, and specific aspects of evaluation and reflection. We then assessed criterion validity of all three models using self-reported skill level.

Table 6 summarizes findings across all models for psychometric and criterion validity statistics. Although fit indices were similar across all models, we conclude that the *refined* model has the most support. Psychometrically, it showed the best convergent and divergent validity of the models, and particularly improved upon the multi-collinearity between subscales seen in the *initial* model. The refined model builds on the foundations laid by Toering et al.'s (2012a) SRL-SRS and Bartulovic et al.'s (2017) SPV and further maintains conceptual distinctions between subscales as much as possible. With these advantages, we also noted that the criterion validity effect sizes (partial eta squared values) for the refined model were not substantially lower than those of the initial model, and that skill groups differences for *reflecting* observed in the initial model were maintained with the *evaluating/reflecting* subscale in the refined model.

Despite our efforts to encompass greater self-regulatory breadth with the *extended* model, we did not observe sufficiently favorable evidence to conclude that it is preferable to the *refined* model. Although the extended model had the best model fit indices, all models had acceptable fit; however, the extended model had the lowest effect sizes for criterion validity. Further, our intent in creating an extended inventory of items was to include concepts highlighted by SRL theory, such as goal setting, that were not covered in Toering et al.'s (2012a) SRL-SRS. However, as we tested this item catalog with increasing conceptual breadth, we had greater difficulty maintaining the distinction between subscales. In the end, the extended model ironically had only four subscales. Further, the only significant skill group differences for the extended model pertained to motivational subscales, with no differences on any metacognitive subscale. We believe this is problematic because any SRL assessment should capably discriminate performers on motivational *and* metacognitive facets; thus, advocacy for our extended model would be limited in terms of conceptual validity and would curtail future research.

Our interests lie not only in researching and understanding the conceptual role of SRL in sport, but eventually in supporting athletes, coaches, and mental performance consultants to effectively assess SRL processes in their practice. As such, a shorter, more practical version has appeal. The 26 items in the refined model is a substantial reduction from Toering et al.'s (2012a) 46 items, and an additional reduction relative to Bartulovic et al.'s (2017) 31 items. Thus, an additional merit of the *refined* model over the others was that it retained fewer items. Altogether, considering practical reasons, factorial and criterion validity, we recommend the refined model or what we now term the Self-Regulated Learning Scale—Sport Practice (SRL-SP) for use in future work by researchers in the field of expert development and optimal practice conditions.

### Further Development for the SRL-SP

The SRL-SP could possibly withstand further reduction of items; for instance, the nine items for *planning* demonstrate substantial redundancy. Although it is beyond the scope of this manuscript, we see possibilities for creating a shorter version of the SRL-SP that can be used for both research and practice. A shorter version may also present the possibility of a composite SRL score, rather than a collection of subscales. Indeed the fact that the number of subscales decreased in each of our subsequent measurement models led us to question whether it was realistic to maintain distinct subscales for subprocesses subsumed under SRL given that SRL is cyclical and dynamic, with subprocesses that are interwoven by definition. Future research is needed to understand the optimal weighting of each subscale toward a composite score and whether this score would provide value in terms of factor and criterion validity beyond subscale scores.

Our criterion validity analyses focused on skill group differences, however, this is only one criterion hallmark of research within expert development frameworks. The validity of SRL-SP scores must be tested against the second proposition, i.e., *associations with practice* (Ericsson and Smith, 1991; e.g., Abernethy et al., 1993; Tedesqui and Young, 2017). As optimization of learning efforts and practice activities are predicated upon enactment of SRL processes (McCardle et al., 2017), future research should examine associations between SRL-SP scores and measures of sport-specific practice, and ideally with amounts and qualities of deliberate practice (Ericsson et al., 1993). Importantly, if research is to inform expert *development*, it essential to examine SRL-SP scores and their associations with skill level and practice longitudinally.

More research is needed to understand the application and impact of SRL as athletes develop. In terms of the limitations of self-report measurement, athletes of different skill levels or ages may interpret SRL-SP items differently. This may have had a bearing on why SRL-SP did not discriminate skill levels among junior athletes. There may also be differences in the facility with which athletes at different positions across the spectrum of skill development consciously report their enactment of SRL. For example, a surprising pattern emerged in the current study where local/regional athletes often reported higher levels of SRL engagement than national and provincial athletes (see Figure 1). One hypothesis could be that SRL is more consciously applied and reported when lower skill athletes (e.g., local/regional) engage in a lot of skill acquisition, or when the highest-level athletes who have sophisticated understanding of their sport task requirements are engaging in targeted skill refinement. It may be that mid-spectrum (e.g., national or provincial) athletes use SRL subprocesses, but either not as consciously or apply them more selectively, meaning they would be report less use of SRL.

Future work should consider examining the convergent validity of the SRL-SP with other indicators of SRL. Athletes' SRL-SP scores could be compared to observable indicators of metacognition and motivation (Toering et al., 2011), and/or triangulated with coach, teammate or parental ratings of SRL. Microanalytic approaches (e.g., Cleary and Zimmerman, 2001) and think aloud protocols (e.g., Coughlan et al., 2013) relating to *in situ* practice scenarios could also be used for cross-validation purposes.

## Conclusion

Our aim was to advance development of a SRL instrument for further research and, in the long-term, eventual use in practice. We tested three survey catalogs and ultimately chose a refined model, the SRL-SP survey, which has five subscales: planning, checking, evaluating/reflecting, SEC, and effort. As researchers explore factors that contribute to athletes' development toward sport expertise, SRL has been highlighted as a variable that impacts the quantity and quality of deliberate practice (Elferink-Gemser et al., 2015; Tedesqui and Young, 2015; McCardle et al., 2017; Baker et al., 2018). The SRL-SP may represent a step forward in assessing athletes' self-processes associated with the optimization of sport practice, from which self-report scores might be used to distinguish athletes who tend to “go through the motions” during training, from those athletes who tend to deliberately design, consciously engage in, and strategically refine their activities.

## Ethics Statement

This study was carried out in accordance with the recommendations of Ethical Conduct for Research Involving Humans, Social Science and Humanities Research Council of Canada with written informed consent from all subjects. All subjects gave written informed consent in accordance with the Declaration of Helsinki. The protocol was approved by the University of Ottawa Health Sciences and Science Research Ethics Board.

## Author Contributions

LM, BY, and JB contributed to the conception and design of the study. LM performed the statistical analysis; all authors contributed to interpretation. LM wrote the first draft of the manuscript and all authors contributed to the revision of the manuscript. All authors edited and gave final approval for publication and were accountable for this work.

### Conflict of Interest Statement

The authors declare that the research was conducted in the absence of any commercial or financial relationships that could be construed as a potential conflict of interest.
