# Supplementary material for: Two-Phase Evaluation of the Validity of a Measure for Self-Regulated Learning in Sport Practice
Source: Front Psychol. 2018 Dec 21;9:2641. doi: 10.3389/fpsyg.2018.02641 (PMC6309132; doi:10.3389/fpsyg.2018.02641)
Supplement: Supplementary file 1 [file Table_1.DOCX]

**Appendix A**

EFA Iterations and Item Removal Decisions for *Refined Model*

|  |  | Model Fit Indices | | | | Item |  |
| --- | --- | --- | --- | --- | --- | --- | --- |
| EFA | Items | Model | EV^a^ | CFI | TLI | Removed | Rationale^b^ |
| 1 | 35 | 8-factor | 7 | .939 | .895 | 29 | Loaded on factor alone; no other loadings > .3; no other *r* > .5 |
|  |  |  |  |  |  | 09 | No loadings > .3; no *r* > .5 |
|  |  |  |  |  |  | 14 | No loadings > .35; no *r* > .35 |
| 2 | 32 | 6-factor | 7 | .933 | .896 | 51 | No loadings > .3; no *r* > .5; cross-loading < .2 |
|  |  |  |  |  |  | 45 | No loadings > .31; no *r* > .4; cross-loading < .2 |
| 3 | 30 | 5-factor | 7 | .932 | .900 | 26 | No loadings > .3; no *r* > .5; cross-loading < .2 |
| 4 | 29 | 5-factor | 6 | .940 | .910 | 08 | No loadings > .3; no *r* > .51; cross-loading < .2 |
| 5 | 28 | 5-factor | 6 | .946 | .911 | 05 | No loadings > .3; no *r* > .5; cross-loading < .2 |
| 6 | 27 | 5-factor | 5 | .950 | .923 | 32 | Cross-loading < .2; no loadings > .4 |
| 7 | 26 | 5-factor | 5 | .948 | .918 |  |  |

a Number of factors with Eigenvalues above 1.

b Conceptual rationale was always considered.

**Appendix B**

EFA Iterations and Item Removal Decisions for *Extended Model*

|  |  | Model Fit Indices | | | | Item |  |
| --- | --- | --- | --- | --- | --- | --- | --- |
| EFA | Items | Model | EV^a^ | CFI | TLI | Removed | Rationale^b^ |
| 1 | 47 | 7-factor | 8 | .911 | .876 | 05 | No loadings > .4; only 1 *r* > .5; cross-loading < .2 |
|  |  |  |  |  |  | 08 | No loadings > .4; *r* > .6 with 3 factors; cross-loading < .2 |
|  |  |  |  |  |  | 21 | Cross-loading < .2; *r* > .6 with 2 factors |
|  |  |  |  |  |  | 24 | Loaded on factor alone; *r* > .5 with 3 factors; cross-loading < .2 |
|  |  |  |  |  |  | 28 | Cross-loading < .2; *r* > .6 with 2 factors |
|  |  |  |  |  |  | 51 | No loadings > .4; only 1 *r* > .5; cross-loading < .2 |
|  |  |  |  |  |  | 52 | No loadings > .4; only 1 *r* > .5; cross-loading < .2 |
| 2 | 40 | 6-factor | 6 | .934 | .907 | 14 | No loadings > .4; no *r* > .5 |
|  |  |  |  |  |  | 29 | No loadings > .4; no *r* > .5 |
|  |  |  |  |  |  | 40 | No loadings > .4; no *r* > .5 |
|  |  |  |  |  |  | 45 | No loadings > .4; no *r* > .5 |
| 3 | 36 | 6-factor | 5 | .938 | .908 | 09 | No loadings > .4; *r* > .6 with 2 factors; cross-loading < .2 |
|  |  |  |  |  |  | 17 | No loadings > .4; *r* > .6 with 2 factors; cross-loading < .2 |
|  |  |  |  |  |  | 23 | No loadings > .4; *r* < .6; cross-loading < .2 |
|  |  |  |  |  |  | 33 | No loadings > .4; cross- loading < .01 |
| 4 | 32 | 5-factor | 5 | .938 | .912 | 15 | Conceptual only; highest loading item on *checking* factor |
| 5 | 31 | 4-factor | 4 | .945 | .926 | 06 | No loadings > .4; only 1 *r* > .5; cross-loading < .2 |
| 6 |  |  |  |  |  | 23 | No loadings > .4; *r* > .55 with 2 factors; cross-loading < .2 |
| 6 | 29 | 4-factor | 4 | .946 | .928 |  |  |

a Number of factors with Eigenvalues above 1.

b Conceptual rationale was always considered.
